# Ocular Vestibular-Evoked Myogenic Potential Amplitudes Elicited at 4 kHz Optimize Detection of Superior Semicircular Canal Dehiscence

**DOI:** 10.3389/fneur.2020.00879

**Published:** 2020-08-25

**Authors:** Emma D. Tran, Austin Swanson, Jeffrey D. Sharon, Yona Vaisbuch, Nikolas H. Blevins, Matthew B. Fitzgerald, Kristen K. Steenerson

**Affiliations:** ^1^Department of Otolaryngology—Head and Neck Surgery, Stanford University School of Medicine, Stanford, CA, United States; ^2^Department of Otolaryngology—Head and Neck Surgery, University of California, San Francisco, San Francisco, CA, United States; ^3^Department of Otolaryngology—Head and Neck Surgery, Rambam Medical Center, Haifa, Israel

**Keywords:** superior semicircular canal dehiscence, vestibular evoked myogenic potential, vestibular testing, vestibular dysfunction, third window, computed tomography, temporal bone

## Abstract

**Introduction:** High-resolution temporal bone computed tomography (CT) is considered the gold standard for diagnosing superior semicircular canal dehiscence (SCD). However, CT has been shown over-detect SCD and provide results that may not align with patient-reported symptoms. Ocular vestibular-evoked myogenic potentials (oVEMPs)—most commonly conducted at 500 Hz stimulation—are increasingly used to support the diagnosis and management of SCD. Previous research reported that stimulation at higher frequencies such as 4 kHz can have near-perfect sensitivity and specificity in detecting radiographic SCD. With a larger cohort, we seek to understand the sensitivity and specificity of 4 kHz oVEMPs for detecting clinically significant SCD, as well as subgroups of radiographic, symptomatic, and surgical SCD. We also investigate whether assessing the 4 kHz oVEMP n10-p15 amplitude rather than the binary n10 response alone would optimize the detection of SCD.

**Methods:** We conducted a cross-sectional study of patients who have undergone oVEMP testing at 4 kHz. Using the diagnostic criteria proposed by Ward et al., patients were determined to have SCD if dehiscence was confirmed on temporal bone CT by two reviewers, patient-reported characteristic symptoms, and if they had at least one positive vestibular or audiometric test suggestive of SCD. Receiver operating characteristic (ROC) analysis was conducted to identify the optimal 4 kHz oVEMP amplitude cut-off. Comparison of 4 kHz oVEMP amplitude across radiographic, symptomatic, and surgical SCD subgroups was conducted using the Mann-Whitney U test.

**Results:** Nine hundred two patients (*n*, ears = 1,804) underwent 4 kHz oVEMP testing. After evaluating 150 temporal bone CTs, we identified 49 patients (*n*, ears = 61) who had radiographic SCD. Of those, 33 patients (*n*, ears = 37) were determined to have clinically significant SCD. For this study cohort, 4 kHz oVEMP responses had a sensitivity of 86.5% and a specificity of 87.8%. ROC analysis demonstrated that accounting for the inter-amplitude of 4 kHz oVEMP was more accurate in detecting SCD than the presence of n10 response alone (AUC 91 vs. 87%). Additionally, using an amplitude cut-off of 15uV reduces false positive results and improves specificity to 96.8%. Assessing 4 kHz oVEMP response across SCD subgroups demonstrated that surgical and symptomatic SCD cases had significantly higher amplitudes, while radiographic SCD cases without characteristic symptoms had similar amplitudes compared to cases without evidence of SCD.

**Conclusion:** Our results suggest that accounting for 4 kHz oVEMP amplitude can improve detection of SCD compared to the binary presence of n10 response. The 4 kHz oVEMP amplitude cut-off that maximizes sensitivity and specificity for our cohort is 15 uV. Our results also suggest that 4 kHz oVEMP amplitudes align better with symptomatic SCD cases compared to cases in which there is radiographic SCD but no characteristic symptoms.

## Introduction

In 1998, Minor et al. reported on a series of difficult-to-diagnose patients who experienced sound- or pressure-induced vertigo and demonstrated nystagmus in the plane of the superior semicircular canal (SSC) (1). On computed tomography (CT) imaging, they were found to have a bony dehiscence above the SSC, which was confirmed through surgical exploration and repair. These patients were diagnosed with superior semicircular canal dehiscence (SCD). The opening between the inner ear and cranial cavity creates a novel low-impedance pathway, which re-routes some of the acoustic energy generated from the middle ear to the labyrinth. This phenomenon was described as a third-window effect (2) to which the classic constellation of SCD symptoms (e.g., bone-conduction hyperacusis, pulsatile tinnitus, and sound- or pressure-induced vertigo) is attributed (3, 4).

Since its discovery, SCD has posed a great diagnostic challenge. Identification of the dehiscence on high-resolution temporal bone CT has long been the gold standard of diagnosis but has remained limited by the variability of CT scanner quality, imaging protocols, and interpretations (5). Even with sub-millimeter resolutions, CT scans may still be unable to visualize very thin bone (6). This has led to the radiographic prevalence of SCD (7–9) being considerably higher than those found in cadaveric temporal bone studies (10). In addition, it is thought that many patients with SCD can be asymptomatic or can present with non-specific symptoms potentially related to other etiologies (11). Other vestibular disorders that cause dizziness, including migraine, are often seen in patients with SCD (12, 13). Some are thought to be “sensitized” by SCD or can just occur concomitantly (14). Given that imaging can over-detect dehiscence and SCD symptoms can present variably, Ward et al. proposed diagnostic criteria for clinically significant SCD (hereafter referred to as SCD_θ_), which required both evidence on CT and specific symptoms characteristic of SCD, as well as a third criterion of a positive finding on physiologic testing. The third criterion can be valuable in the diagnosis and management of SCD when there is uncertainty regarding imaging or symptoms.

Vestibular-evoked myogenic potentials (VEMPs) were first described in 1994 (15). They are thought to reflect a reflex arc in which stimulation of the saccule and utricle generate a myogenic response in the ipsilateral sternocleidomastoid (i.e., cervical or cVEMPs) (15) or the contralateral inferior oblique (i.e., ocular or oVEMPs), respectively (16–18). These organs normally respond to loud acoustic stimuli, but in the setting of a third window, responses are exaggerated (19, 20). Unsurprisingly, then, VEMPs have become an increasingly important part of the diagnostic battery for SCD. Lower cVEMP threshold was the first to be reported to correlate with radiographic SCD (21, 22), and then later, oVEMP amplitude was shown to correlate better with surgically confirmed SCD (23, 24). However, given the variability of these tests due to factors such as age; degree of conductive hearing loss; and even testing equipment, operators, and protocols (25–27); guidelines for incorporating these tests into the diagnostic battery remain ambiguous (28). Manzari et al. demonstrated that the binary presence of the oVEMP n10 response stimulated at a higher frequency such as 4 kHz had a sensitivity and specificity of 100% in 22 patients with radiographic SCD (29). Lin et al. recently validated the superior diagnostic accuracy of 4 kHz oVEMPs n10 response in a similar but larger patient population. However, they were unable to attain the perfect sensitivity and specificity seen by Lin et al. (30).

In this study, we seek to assess the performance of 4 kHz oVEMP in detecting clinically significant SCD_θ_, as defined by the Ward et al. diagnostic criteria (28), in addition to detecting subgroups of radiographic, symptomatic, and surgical SCD. Given the number of clinical false positives, we also seek to determine whether assessing the amplitude rather than the binary presence of the n10 response for 4 kHz oVEMP can further optimize the detection of SCD.

## Methods

### Subjects

We conducted a cross-sectional study of patients seen at our tertiary referral center and who underwent vestibular testing between October 2016 and October 2019. Patients with oVEMP testing conducted at 500 Hz and 4 kHz, cVEMP testing conducted at 500 Hz, audiometric testing, high-resolution computed tomography (CT) imaging, and clinical data including symptomatology were included in the analysis. Study subjects were excluded if they (1) did not have reliable CT imaging studies, (2) had no measurable response for both cVEMP and oVEMP testing, (3) had 4 kHz oVEMP waveforms that were non-reproducible or lacked either a discernable n10 trough or p15 peak, (4) had abnormal tympanometry, or (5) had a history of ear surgeries or middle-ear conditions that could negate the VEMP response.

Our study cohort was defined by the diagnostic criteria for SCD proposed by Ward et al. (SCD_θ_), which includes: (1) dehiscence identified on high-resolution CT; (2) at least one of the following characteristic symptoms: autophony or hyperacusis, sound- or pressure-induced vertigo, or pulsatile tinnitus; and (3) at least one of the following audiometric test results: negative bone conduction thresholds on pure-tone audiometry, low cVEMP thresholds, or high oVEMP amplitudes (28). A subject was considered to have negative bone conduction when thresholds were <0 db HL at any frequency. A high oVEMP amplitude was defined as peak-to-peak measurements ≥17 uV at 500 Hz stimulus (23). A cVEMP threshold was considered low when the lowest intensity 500 Hz stimulus that could elicit a reproducible characteristic p13 n23 waveform was ≤75 dB nHL. While third criterion proposed by Ward et al. includes other VEMP testing that may correlate with 4 kHz oVEMP results, these tests are still believed to represent independent physiological responses to different acoustic stimuli. Therefore, we included all three criteria when defining our study cohort.

Through a retrospective chart review, radiographic dehiscence was considered confirmed when both the reading neuro-radiologist and diagnosing physician identified a dehiscence on CT imaging. If there was a discrepancy between this initial review of the CT, three expert reviewers (consisting of two neurotologists and one otoneurologist) adjudicated the results through a tie-break protocol. Images that demonstrate thinning or near-dehiscence of the temporal bone were considered negative for SCD. Subjects were considered symptomatic if they had positive dehiscence on CT, as defined by the criteria above; demonstrated symptoms that are characteristic of SCD; and the symptoms were determined to be related to the dehiscence by the diagnosing physician.

### Audiometric Procedures

Audiometric data were obtained as part of audiologic evaluations at our institution's audiology clinic. Tests were completed in a double-walled sound booth using GSI Audiostar Pro (Grason-Stadler) audiometers and conducted using ER-3A insert earphones or Sennheiser HDA 200 circumaural headphones. A modified Hughson-Westlake method was used to measure air-conduction and bone-conduction thresholds, which could be measured as low as −10 dB HL (31). Bone-conduction testing was conducted with masking if the difference between air- and unmasked bone-conduction thresholds were >10 dB HL. Air-bone gap averages were calculated using the average of the frequencies 250, 500, and 1,000 Hz (32, 33).

### VEMP Testing

VEMP testing was completed using an Intelligent Hearing Systems Smart USB (Intelligent Hearing Systems, 6860 SW 81st Street Miami, FL 33143, USA) evoked potential system. Ipsilateral cVEMP results were obtained with the patient reclined to 30 degrees above horizontal, with the head rotated 45 degrees from the test ear, and held above the exam throughout each run. Contralateral oVEMP recordings were obtained with the patient seated upright with head position held level and eye gaze held 30 degrees above horizontal. Air conduction 500 Hz tone bursts were used as stimuli for cVEMP and oVEMP threshold search and inter-amplitude measurement for each ear. Air conduction 4 kHz tone bursts delivered at 95 dB nHL were also used as stimuli for measurement of oVEMP inter-amplitude for each ear. Stimulus envelope characteristics for all VEMP stimuli had a rise, plateau, and fall of 2, 1, and 2 ms, respectively. Amplifier gain was set to 5,000 for cVEMP and 100,000 for oVEMP. The cVEMP high-pass and low-pass filter was set to 10 and 1,500 Hz, respectively. The oVEMP high-pass and low-pass filter was set to 1 and 1,000 Hz, respectively. For threshold search, stimulus intensity was decreased in 10 dB steps until threshold was obtained as the last reproducible response. An evoked potential response is defined as the presence of a reproducible n1 negative peak. The highest inter-amplitude between the n1 and p1 negative and positive peaks, respectively, were manually measured using the software interface. All audiometric data including pure-tone and VEMP testing were prospectively recorded in a custom relational database in Filemaker (Claris International Inc., Santa Clara, CA, USA).

### Analysis

R version 3.6.1 (R Foundation for Statistical Computing, Vienna, Austria) (34) was used for statistical analysis with the finalfit (35) package for generation of data tables, ggplot2 (36) package for data visualization, and pROC (37) packages for receiver operating characteristic (ROC) analysis. For the demographics table, we used the Mann-Whitney *U*-test for continuous variables and Pearson's chi-squared test or Fisher's exact test for categorical variables, as appropriate. To identify the optimal diagnostic cut-off for 4 kHz oVEMP amplitude, we conducted a ROC analysis. Sensitivity and specificity were calculated for each amplitude cut-off and the associated 95% confidence interval (CI) are “exact” Clopper-Pearson intervals. Area under the curve was calculated for each cut-off and compared using DeLong's test. Comparisons of 4 kHz oVEMP amplitude across SCD subgroups, as well as characteristic symptoms, were conducted using the Mann-Whitney U test. All results were considered significant at α = 0.05.

## Results

### Demographics

We identified 1,168 patients (*n*, ears = 2,367) who underwent 500 Hz oVEMP testing and, of those, 902 patients (*n*, ears = 1,804) who also underwent 4 kHz oVEMP testing. High-resolution temporal bone CT scans were available for 150 patients (*n*, ears = 300) for each of whom a detailed chart review was conducted. Four patients (*n*, ears = 6) underwent bilateral SCD repair and were excluded due to lack of pre-operative 4 kHz oVEMP testing. Thirty-three (33) ears were excluded for previous ear surgeries, including 6 ears for unilateral SCD repair without pre-operative 4 kHz oVEMP. Sixty-three (63) ears were excluded due to failure in eliciting any cVEMP or oVEMP responses. Two ears were excluded for abnormal tympanometry.

**Figure 1:**
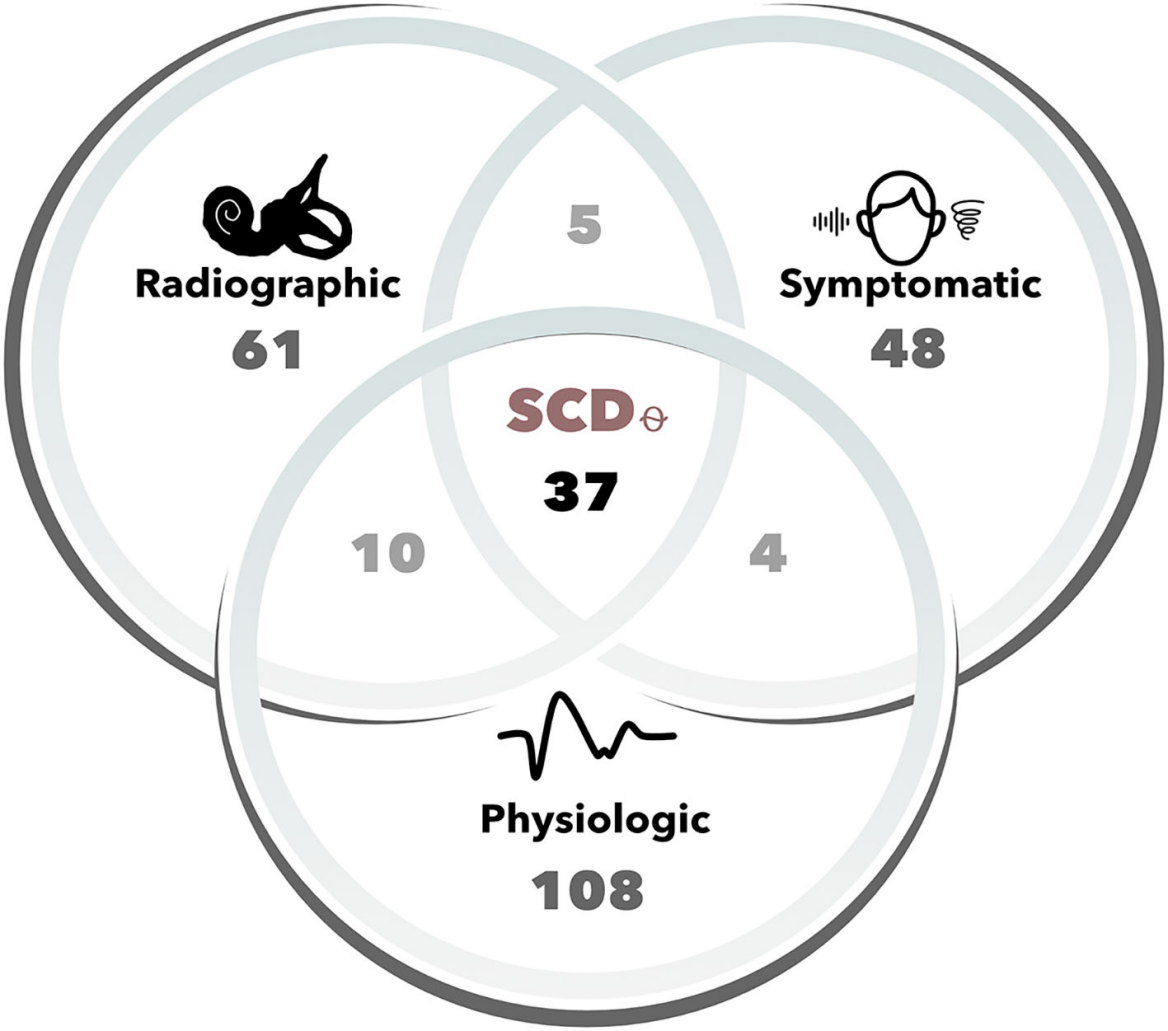
Number of cases for each Ward et al. diagnostic criteria of SCD_θ_. Cases (i.e., ears) were determined to be radiographically positive based upon two-party review of high-resolution (sub-millimeter) temporal bone computed tomography (CT) scans. Symptomatic cases included one of the following patient-reported symptoms: hyperacusis/autophony, pulsatile tinnitus, sound-, or pressure-induced vertigo. Physiologic cases were those that had either 500 Hz oVEMP amplitude ≥ 17 uV, 500 Hz cVEMP threshold ≤ 75 dB nHL, or negative bone-conduction thresholds. SCD_θ_ refers to our study cohort of clinically significant SCD, as defined by the radiographic, symptomatic, and physiologic diagnostic criteria proposed by Ward et al.

In total, 49 patients (*n*, ears = 61) were found to have radiographic dehiscence on CT, 44 patients (*n*, ears = 48) reported symptoms characteristic of SCD, and 69 patients (*n*, ears = 108) had positive VEMP findings (Figure 1). Of these, 33 patients (*n*, ears = 37) met all three Ward et al. criteria for SCD_θ_. Patient demographic, audiometric and vestibular testing features are shown for SCD_θ_ patients and controls in Table 1.

**Table 1:** Patient characteristics for patients with superior semicircular canal dehiscence.

		**SCD_**θ**_**	**Control**	**Total**	***p***
Age (%)	<40	7 (18.9)	49 (31.4)	56 (29.0)	0.285
	40–49	12 (32.4)	32 (20.5)	44 (22.8)	
	50–59	9 (24.3)	43 (27.6)	52 (26.9)	
	≥60	9 (24.3)	32 (20.5)	41 (21.2)	
Sex (%)	F	25 (67.6)	108 (69.2)	133 (68.9)	0.844
	M	12 (32.4)	48 (30.8)	60 (31.1)	
Radiographic dehiscence (%)	Absent	0 (0.0)	132 (84.6)	132 (68.4)	<0.001
	Present	37 (100.0)	24 (15.4)	61 (31.6)	
Characteristic symptoms (%)	Absent	0 (0.0)	145 (92.9)	145 (75.1)	<0.001
	Present	37 (100.0)	11 (7.1)	48 (24.9)	
Surgical repair (%)	Not Repaired	27 (73.0)	154 (98.7)	181 (93.8)	<0.001
	Repaired	10 (27.0)	2 (1.3)	12 (6.2)	
500 Hz cVEMP Threshold (dB nHL)	Median (IQR)	75.0 (10.0)	90.0 (15.0)	90.0 (15.0)	<0.001
500 Hz oVEMP Amplitude (uV)	Median (IQR)	88.5 (55.4)	12.5 (23.2)	15.2 (34.3)	<0.001
4 kHz oVEMP Amplitude (uV)	Median (IQR)	23.0 (15.9)	0.0 (0.0)	0.0 (7.5)	<0.001
Negative bone conduction thresholds (%)	Absent	21 (58.3)	126 (84.0)	147 (79.0)	0.001
	Present	15 (41.7)	24 (16.0)	39 (21.0)	
Air-Bone Gap at 250, 500, 1,000 Hz (dB HL)	Median (IQR)	10.8 (14.2)	5.0 (7.5)	5.0 (6.7)	<0.001

*SCD_θ_ refers to our study cohort of clinically significant SCD, as defined by the radiographic, symptomatic, and physiologic diagnostic criteria proposed by Ward et al. Our control cohort are individuals who do not meet these diagnostic criteria*.

*SCD, superior semicircular canal dehiscence; dB, decibels; nHL, normal hearing loss; HL, hearing loss; F, female; M, male*.

### 4 kHz oVEMP and Superior Semicircular Canal Dehiscence

Of the 193 cases (i.e., ears) reviewed, 51 had a positive 4 kHz oVEMP n10 response. There were a total of 37 cases with SCD_θ_ and 157 without SCD_θ_. Table 2 presents a frequency table of 4 kHz oVEMP n10 response to SCD_θ_. Sensitivity for 4 kHz oVEMP response was 86.5% [95% CI: 71.2, 95.5], with 32 out of 37 SCD_θ_ cases demonstrating an n10 response and 19 non-SCD_θ_ cases that were falsely positive. With an estimated prevalence of 1% based on our institution's clinic data, the positive predictive value (PPV) was 6.7% [95% CI: 4.4, 10.0]. Specificity was calculated as 87.8% [95% CI: 81.6, 92.5], with 137 out of 156 non-SCD_θ_ cases without an n10 response and 5 SCD_θ_ cases that were falsely negative. Negative predictive value (NPV) was 99.8% [95% CI: 99.6, 99.9]. Table 3 summarizes the sensitivity, specificity, PPV, and NPV, and area under the curve (AUC) of 4 kHz oVEMP n10 responses, compared to those of increased 500 Hz oVEMP amplitude and decreased 500 Hz cVEMP thresholds.

**Table 2:** Frequency table for 4 kHz oVEMP n10 response for diagnosis of SCD_θ_.

	**Superior semicircular canal dehiscence**		
	**SCD_**θ**_**	**Control**	**Total**
+ n10 response	32	19	51
– n10 response	5	137	142
Total	37	156	

*SCD_θ_ refers to our study cohort of clinically significant SCD, as defined by the radiographic, symptomatic, and physiologic diagnostic criteria proposed by Ward et al. Our control cohort are individuals who do not meet these diagnostic criteria*.

**Table 3:** VEMP sensitivity and specificity for diagnosis of SCD_θ_.

	**Cut-off**	**Sensitivity (%)**	**Specificity (%)**	**PPV (%)**	**NPV (%)**	**AUC (%)**
	**(uV)**	**[95% CI]**	**[95% CI]**	**[95% CI]**	**[95% CI]**	**[95% CI]**
4 kHz oVEMP n10 response	>0 uV	86.5 [71.2, 95.5]	87.8 [81.6, 92.5]	6.7 [4.4, 10.0]	99.8 [99.6, 99.9]	87.1 [81.0, 93.3]
500 Hz oVEMP amplitude	≥17 uV	91.7 [77.5, 98.2]	62.6 [64.5, 70.2]	2.4 [1.9, 3.0]	99.9 [99.6, 100.0]	77.1 [71.2, 83.1]
500 Hz cVEMP threshold	≤75 dB	55.6 [38.1, 72.1]	96.0 [91.5, 98.5]	12.3 [5.7, 24.5]	99.5 [99.3, 99.7]	75.8 [67.4, 84.1]

*SCD, superior semicircular canal dehiscence; PPV, positive predictive value; NPV, negative predictive value; AUC, area under the curve; dB, decibel*.

**Figure 2:**
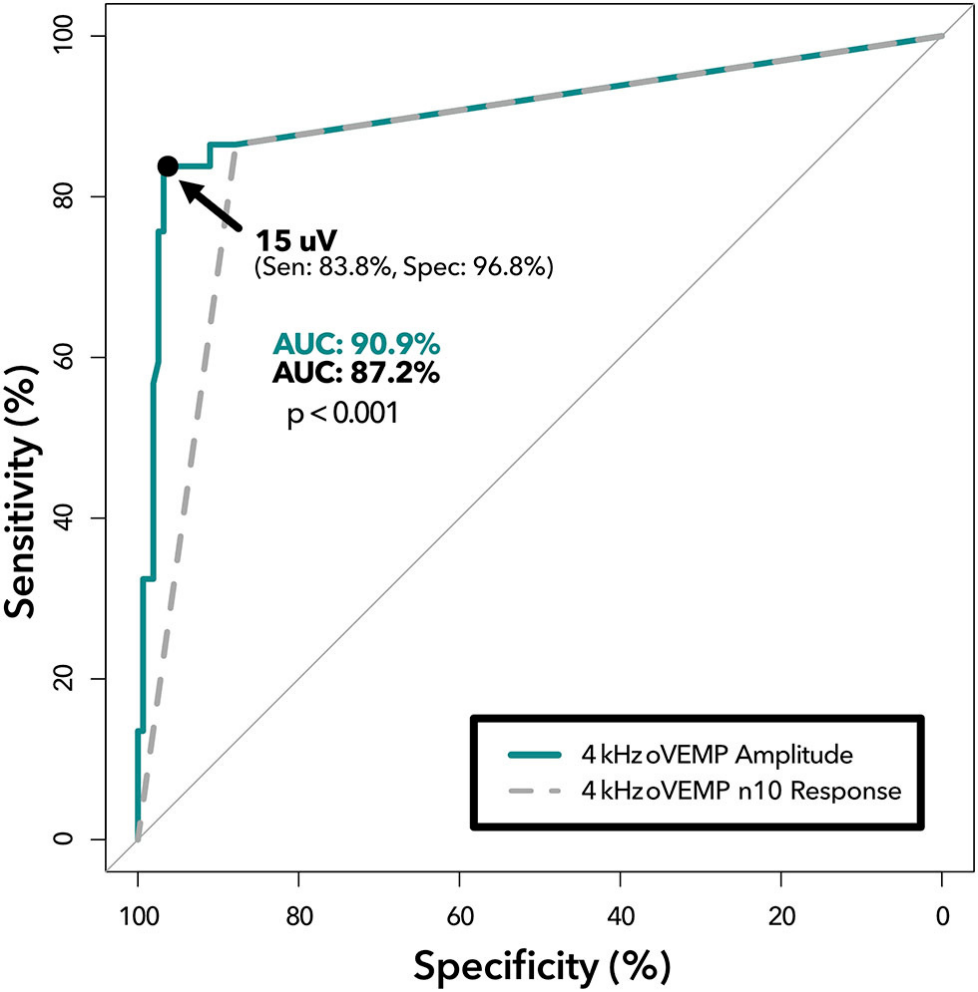
ROC analysis of 4 kHz oVEMP n10 response vs. amplitude. Receiver operating characteristic curves demonstrating diagnostic ability of 4 kHz oVEMP amplitude at various cut-offs (solid line) and 4 kHz oVEMP n10 response (i.e., single amplitude cut-off at >0 uV) (dashed line). The area under the curve (AUC) quantifies the accuracy of detecting SCD for amplitude (top) and n10 response (bottom) with a *p* < 0.001 suggesting significant difference. The data point on the solid line represents the optimal threshold (15 uV) with (sensitivity, specificity) listed.

**Figure 3:**
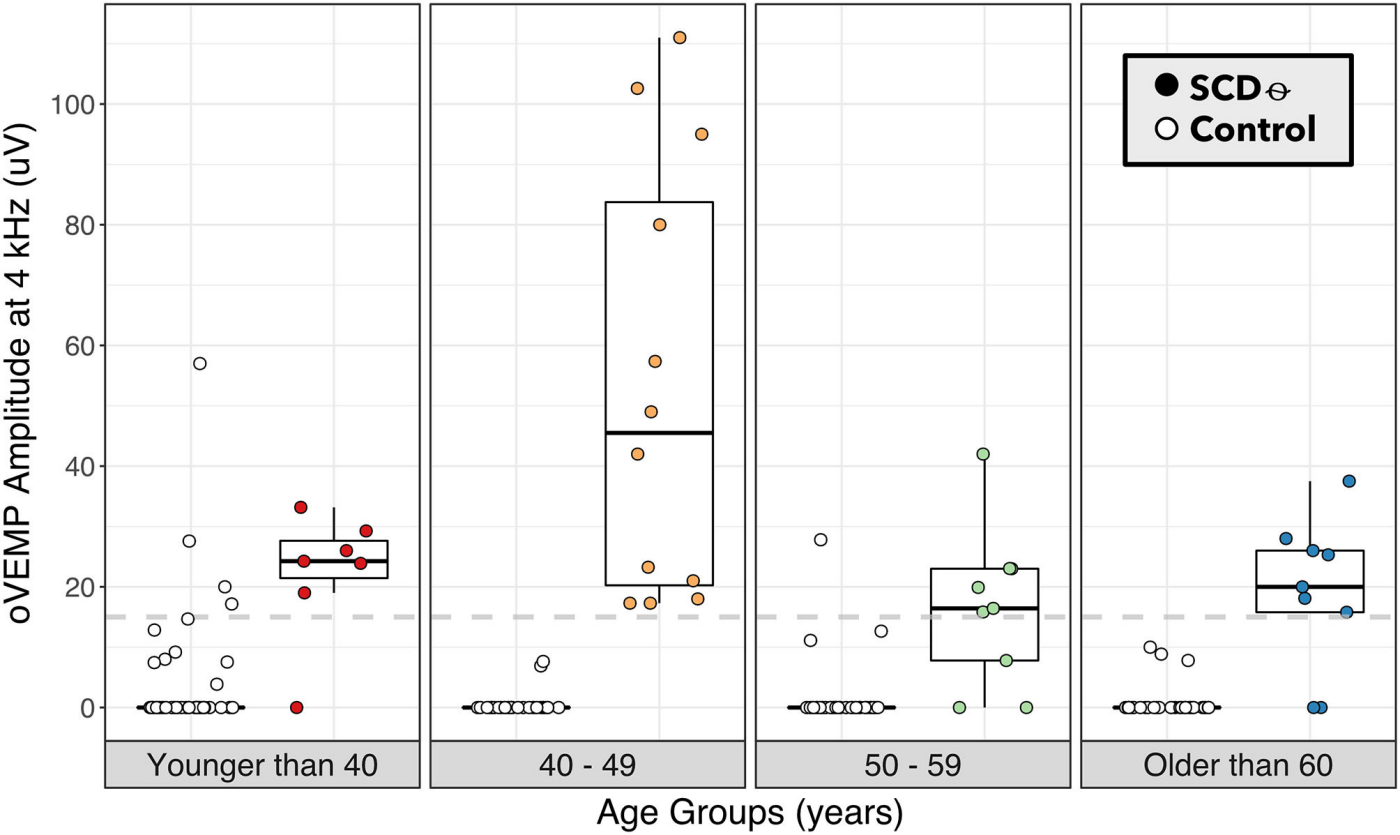
4 kHz oVEMP amplitude binned by age category for SCD_θ_ and control. 4 kHz oVEMP amplitudes of patients with SCD_θ_ (closed circles) and without SCD_θ_ (open circles) separated into age categories of <40, 40–49, 50–59, and ≥60 years old. Dashed line represents the proposed 4 kHz oVEMP amplitude cut-off of 15 uV for detecting SCD_θ_.

ROC-AUC analysis comparing binary 4 kHz oVEMP n10 response (AUC = 87.2%) to 4 kHz oVEMP amplitudes (AUC = 91.0%) found that the accuracy when accounting for the amplitudes was significantly higher (*p* < 0.001) (Figure 2). As expected, the 4 kHz oVEMP amplitude for SCD_θ_ cases (median = 23.0 uV, mean = 29.9 uV) is significantly higher than those for non-SCD_θ_ cases (median = 0 uV, mean = 1.8 uV) (*p* < 0.001). However, as demonstrated in Figure 3, there are still 19 false positive cases when only considering the presence of 4 kHz oVEMP n10 response. Eleven (58%) of these false positive cases are patients who are <40 years old. To reduce the number of false positive cases, the cut-off amplitude of 15 uV was identified by the ROC analysis to optimize testing accuracy with a sensitivity of 83.8% [95% CI: 68.0, 93.8] and a specificity of 96.8% [95% CI: 92.7, 99.0]. PPV increased to 20.9% [95% CI 9.9, 38.8] and NPV increased slightly to 99.8% [95% CI: 99.6, 99.9] (Table 4).

**Table 4:** Sensitivity and specificity of various 4 kHz oVEMP amplitude cut-offs for SCD_θ_.

	**Cut-off (uV)**	**Sensitivity (%) [95% CI]**	**Specificity (%) [95% CI]**	**AUC (%) [95% CI]**	**Accuracy (%)**
4 kHz oVEMP amplitude	>0 uV	86.5 [71.2, 95.5]	87.8 [81.6, 92.5]	87.1 [81.0, 93.3]	87.6
	≥10	83.8 [68.0, 93.8]	93.6 [92.7, 99.0]	88.7 [82.4, 95.0]	91.7
	**≥15**	**83.8** [68.0, 93.8]	**96.8** [92.7, 99.0]	**90.3** [84.1, 96.5]	**94.3**
	≥20	59.5 [42.1, 75.2]	97.4 [93.6, 99.3]	78.5 [70.3, 86.6]	90.2

*Sensitivity, specificity, area under the curve (AUC) and accuracy (i.e., percentage of cases correctly classified) are listed for each representative 4 kHz oVEMP amplitude cut-off. AUC and accuracy are highest at the 4 kHz oVEMP amplitude cut-off of 15 uV. Bolded row indicates the sensitivity, specificity, AUC, and accuracy of the optimal 4 kHz oVEMP amplitude cut-off*.

*AUC, area under the curve*.

### 4 kHz oVEMP and SCD Symptomatology

Almost a third of our patients (14 out of 49, 32.7%) demonstrated radiographic dehiscence but had no evidence of characteristic symptoms of SCD in their medical chart. To assess how well 4 kHz oVEMP response aligns with patient-reported symptoms, we split the our cohort into 4 mutually exclusive subgroups: (1) negative radiographic SCD (*n*, ears = 132), (2) radiographic dehiscence without characteristic symptoms or “Radiographic SCD” (*n*, ears = 18), (3) radiographic dehiscence with characteristic symptoms or “Symptomatic SCD” (*n*, ears = 31), and (4) surgically confirmed dehiscence or “Surgical SCD” (*n*, ears = 12), which included two cases that did not meet the Ward et al. physiologic diagnostic criterion for SCD_θ_.

**Figure 4:**
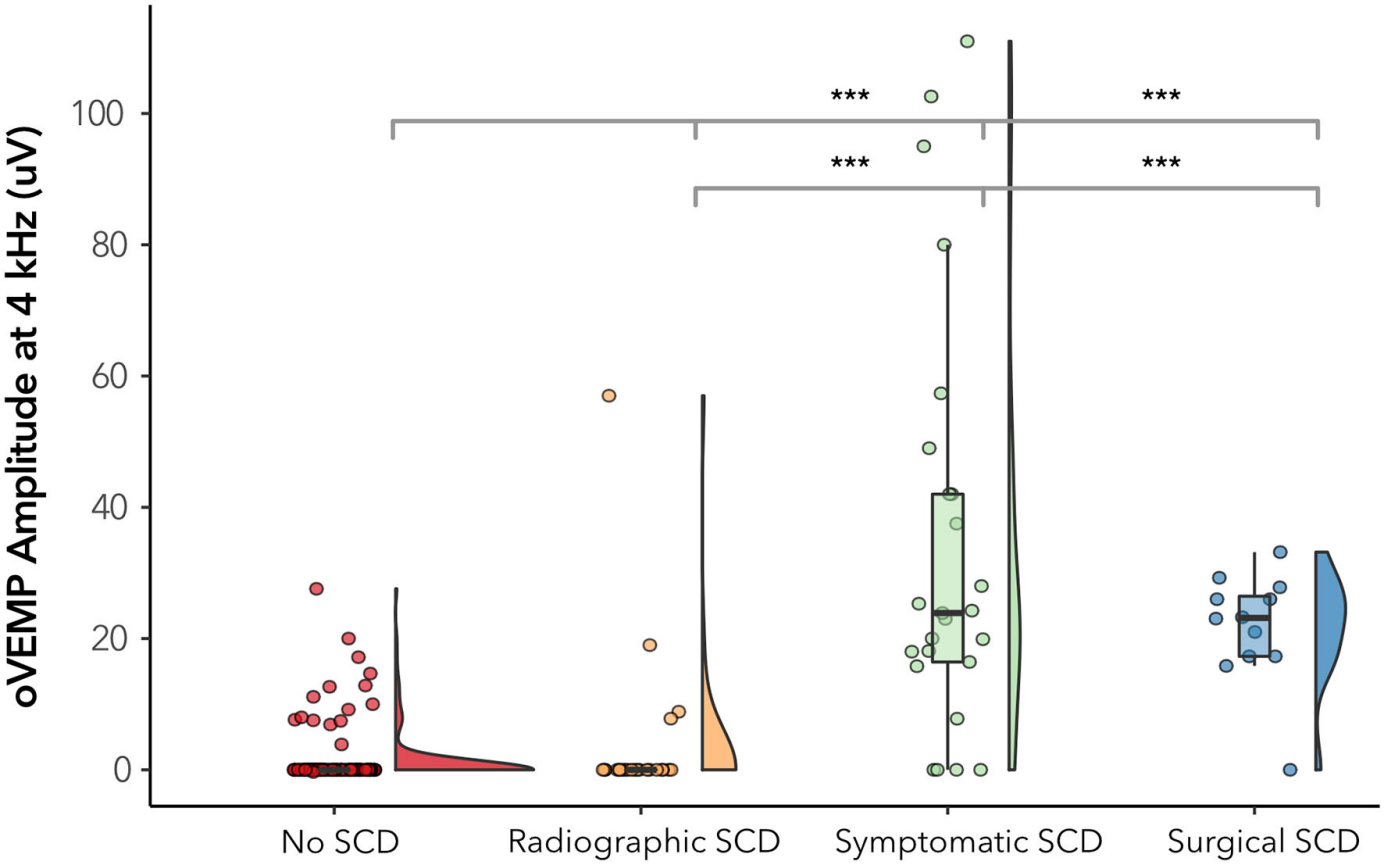
4 kHz oVEMP amplitude across subgroups of SCD. “No SCD” represents cases without any evidence of dehiscence. “Radiographic SCD” are cases with radiographic dehiscence but no characteristic symptoms. “Symptomatic SCD” are cases with radiographic dehiscence and characteristic symptoms. “Surgical SCD” are cases that have undergone surgical repair of dehiscence. The width of the violin diagram depicts the distribution of data. Absence of asterisk (*) indicated *p* > 0.05 and ***p < 0.001.

**Figure 5:**
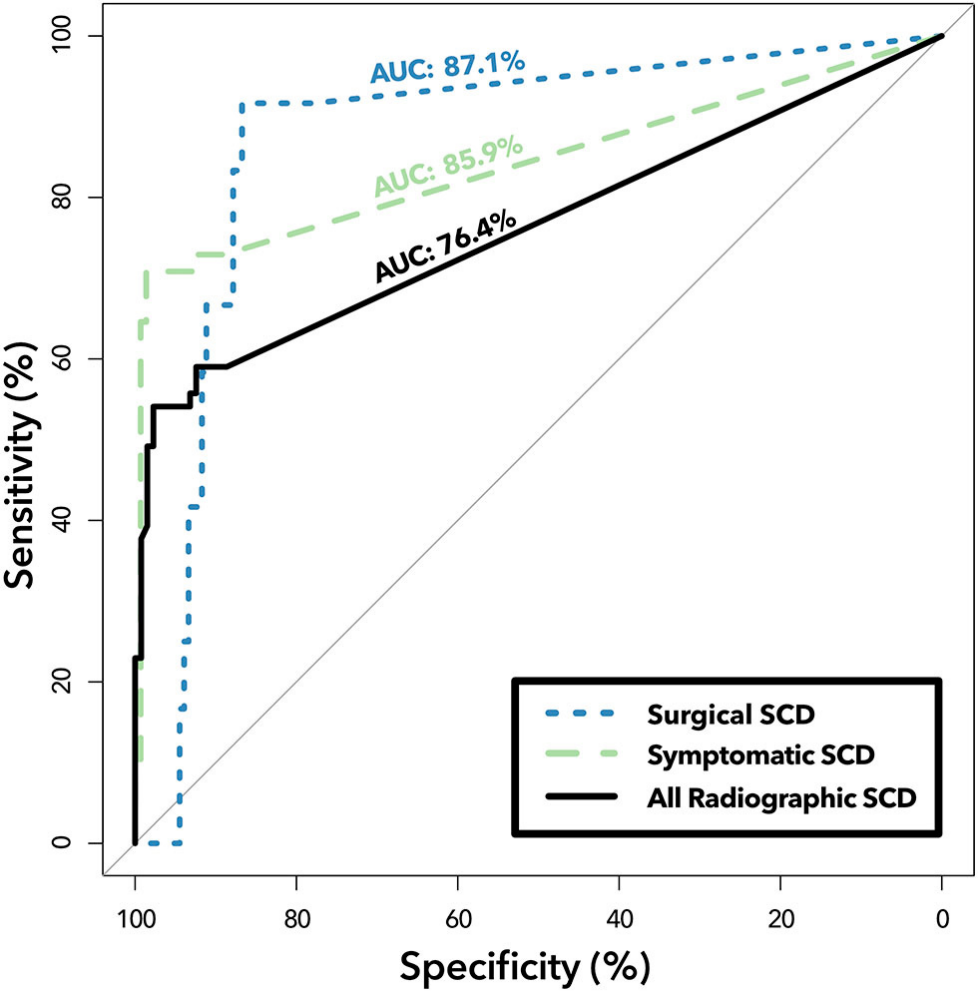
ROC analysis of 4 kHz oVEMP amplitude in detecting subgroups of SCD. Receiver operating characteristic curves demonstrating diagnostic ability of 4 kHz oVEMP amplitude in classifying all cases of radiographic dehiscence (“All Radiographic SCD,” solid line), only symptomatic cases with radiographic dehiscence (“Symptomatic SCD,” dashed line), and surgically repaired cases of SCD (“Surgical SCD,” dotted line). Area under the curve (AUC) quantifies the accuracy for classifying Surgical SCD (top), Symptomatic SCD (middle) and All Radiographic SCD (bottom). The difference between the “All Radiographic SCD” group and the “Symptomatic SCD” group is that patients that have radiographic SCD but without characteristic symptoms are removed from the latter, which improves 4 kHz oVEMP detection performance.

The 4 kHz oVEMP amplitude for symptomatic SCD (median = 19.9 uV, mean = 28.3 uV) and surgical SCD (median = 23.1 uV, mean = 21.7 uV) was significantly higher compared to those without radiographic evidence of SCD (median = 0 uV, mean = 1.34 uV) (*p* < 0.001 for both) (Figure 4). However, the 4 kHz oVEMP amplitude for radiographic SCD (median = 0 uV, mean = 4.09 uV) was similar to those without SCD (*p* = 0.50). The amplitude for 4 kHz oVEMP is significantly higher for patients with symptomatic SCD compared to those with radiographic SCD without characteristic symptoms (*p* < 0.001). The ROC curves in Figure 5 demonstrate the accuracy of 4 kHz oVEMP in detecting all cases of radiographic dehiscence compared to symptomatic and surgical cases.

Comparison of 4 kHz oVEMP amplitudes with symptoms characteristic of SCD demonstrated significantly higher amplitudes associated with aural symptoms of autophony (median 21.5 uV vs. 0 uV, *p* = 0.001) and pulsatile tinnitus (median 21.5 uV vs. 0 uV, *p* = 0.01). Vestibular symptoms such as sound- or pressure-induced vertigo, general vertigo or dizziness, or chronic disequilibrium are not found to be correlated with higher 4 kHz oVEMP amplitudes.

## Discussion

Clinicians consider high-resolution CT imaging and patient-reported symptoms in combination when determining whether a patient could benefit from surgical repair of SCD. This decision, however, can be challenging due to varying quality of CT scanners and techniques, as well as the widely diverse presentation and severity of SCD symptoms that often do not correlate with CT findings (38). Since a positive VEMP result is suggestive of a physiologically active dehiscence, VEMP testing has become a commonly used tool in the testing battery used to support SCD diagnosis and management. Similar to what has been suggested by Manzari et al. and Lin et al., our results demonstrate that 4 kHz oVEMP performs better in detecting SCD than oVEMP amplitudes and cVEMP thresholds elicited at 500 Hz. Though it is not well-understood why higher frequency VEMPs are more specific to SCD, it may be partially due to the otoliths' increased sensitivity to low-frequency sound and vibration. Higher frequency stimuli would therefore be less likely to stimulate the otoliths unless there is a clear third window phenomenon (39). In this study, we sought to optimize the utility of 4 kHz oVEMP in detecting SCD_θ_, as defined by the Ward et al. criteria, by accounting for the amplitude rather than the binary presence of the n10 response alone. We found the optimal threshold for detection was 15 uV for our cohort.

Since its introduction, a wide variety of VEMP cut-offs have been proposed in the literature (23–25, 40, 41). However, offering a cut-off can be challenging given the variability occurring between individuals, and its dependence on age, the degree of conductive hearing loss, and testing operator (25–27). When Manzari et al. first reported that 4 kHz n10 response had 100% accuracy to detecting radiographic SCD; the appeal for this high frequency test was not only in its reported accuracy, but also in the simplicity of a binary, all-or-nothing assessment—something that has eluded 500 Hz testing (29) However, in a larger sample size, our study failed to replicate the perfect sensitivity and specificity previously reported by Manzari et al. for 4 kHz n10 responses. In fact, our detection rate of radiographic SCD was much less with an AUC of 76.4%. This is likely because many of our radiographically positive but asymptomatic patients failed to evoke a 4 kHz oVEMP response. Since Manzari et al. did not address symptomatology in their study cohort, we are unable to make an appropriate comparison.

Similarly, the study by Lin et al. looked at the binary presence of 4 kHz oVEMP n10 response in a larger patient population with radiographic SCD and unspecified symptoms (30). Our analysis of this n10 response compared to SCD found a similar sensitivity to Lin et al. but a lower specificity (88 vs. 93%). This is due to a higher number of false positive cases, which was reduced dramatically when raising the 4 kHz oVEMP amplitude cut-off to 15 uV. By using the amplitude cut-off of 15 uV rather than binary n10 response alone, we were able to improve our specificity to 97% (i.e., a 9% improvement). Given that this is a single institution study, we note that our proposed cut-off may not be completely generalizable, and a large-scale, multicenter study can help to validate this cut-off by accounting for the variability across patient populations, testing device, operators, and protocol. Nevertheless, our results do suggest that evaluating the amplitude of 4 kHz oVEMP response rather than the presence of response alone can improve accurate detection of SCD. In Figure 3, we visualized age categories to explore the well-documented age-related attenuation of VEMP responses. While our small sample size for each age strata limits our ability to propose age-stratified amplitude cut-offs, the results highlight the trend that younger patients have more robust 4 kHz oVEMP responses, which can lead to false positives, and older patients may have more attenuated, but still reliable, 4 kHz oVEMP responses. This challenges the prevailing notion that older patients should not be given VEMP testing (25, 42, 43).

As a tertiary referral center, we see a high volume of potential SCD patients who have complex and non-classical presentations. Even when adhering to strict criteria to develop our study cohort, we still see a relatively high number of SCD_θ_ false positive (*n* = 19) and radiographic false negative (*n* = 24) cases compared to the literature. Review of these cases revealed interesting patterns that may not solely be a limitation of 4 kHz oVEMP testing but can also reflect the complex pathophysiology of SCD. Younger age, by far, seemed to be the most common trait amongst the false positive cases with 11 out of the 19 cases (57.9%) being younger than 40 years old. We also identified 5 of the 19 cases (26.3%) had evidence of Ehlers-Danlos syndrome (EDS), postural orthostatic tachycardia syndrome (POTS), or some other underlying connective tissue disorder. A small case series described SCD and EDS occurring in patients concomitantly (44) This may suggest an association between the two conditions or, perhaps, represent incidental findings in the context that EDS can lead to symptoms and physiological VEMP findings that are similar to SCD but without actual dehiscence. On review of false negative cases (defined as ears with radiographic dehiscence ± symptoms and negative 4 kHz oVEMPs), 14 of 25 cases (56%) were found in bilateral radiographic SCD patients; and 8 (32%) had wide tegmen dehiscence. The wide tegmen dehiscence and bilateral cases can represent extensive disease, which may lead to auto-plugging of the dehiscence by the dura, and a false negative result. This may be more likely given that oVEMP testing is conducted in the upright position (compared to supine with cVEMP testing). Bilateral cases may also have false negative testing if one ear demonstrates a 4 kHz oVEMP response, but the other ear does not—with the more symptomatic ear presumably being more physiologically active. Finally, a few cases had, on average, poorer CT quality or disagreement amongst our CT reviewers. This can call into question whether these cases actually had true dehiscence and may demonstrate the variability in CT quality and interpretation, as well as the tendency for CTs to over-detect dehiscence (5, 11).

Another limitation to our study is that we do not use surgically confirmed SCD to define our study cohort. However, for the subset of 12 surgically confirmed SCD cases, the 4 kHz oVEMP amplitude was shown to be significantly higher than the control. Despite this, our study cohort was defined by radiographic evidence, patient-reported symptoms, and objective test results, which are often used in combination by clinicians to diagnose clinically significant SCD. These diagnostic criteria, however, are not without limitations. For example, 39 cases required additional CT review by our expert panel because the neuro-radiologist and diagnosing physician assessment disagreed or were ambiguous. Many of the patients who underwent 4 kHz oVEMP testing had clinical presentations suggestive of SCD, but by adhering to the strict definition of characteristic symptoms proposed by Ward et al. the number of patients deemed symptomatic was significantly reduced. This suggests a large variability in presenting symptoms for SCD that can go beyond the classical presentation. To define our cohort, we chose 500 Hz oVEMP amplitude and cVEMP threshold cut-offs based upon the literature; however, these cut-offs seem to be much lower than what may be optimal for our patient population and equipment. Despite these limitations, the criteria we used to define our study cohort most accurately reflects the real-life factors clinicians must use in order to determine the patient's surgical candidacy.

We sought to correlate characteristic symptoms collected from chart review with 4 kHz oVEMP amplitudes. This association can be particularly useful in the common situation where presenting symptoms may be multifactorial or due to other comorbidities (38). Some studies have shown that lower cVEMP thresholds were found to correlate with increasing size of dehiscence and higher incidence of vestibular symptoms such as sound-induced vertigo (45, 46). However, the correlation between lower cVEMP thresholds and SCD symptoms have been difficult to reproduce, and many other studies have found that cVEMPs and symptoms do not align (32, 33, 47, 48). Our results suggest that 4 kHz oVEMP amplitudes are significantly higher for patients presenting with classical symptoms of SCD than those patients who have radiographic SCD without these symptoms. One interpretation is that the more physiologically active dehiscences (as measured by the VEMPs) have increased shunting of acoustic energy, leading to more noticeable symptoms. Another interpretation is that some of the patients who are asymptomatic or have atypical symptoms may not actually have dehiscence and instead have a false positive CT and a true negative VEMP. This alignment between 4 kHz oVEMP and symptomatic patients may be useful in helping clinicians to determine surgical candidacy.

Additionally, our results suggest that 4 kHz oVEMP amplitude correlate better with patients who present with aural symptoms like autophony and pulsatile tinnitus, but not with vestibular symptoms such as pressure- or sound-induced vertigo, chronic disequilibrium, or generalized vertigo. Given that 4 kHz oVEMP directly tests the stimulation of vestibular organs, the lack of correlation to sound- and pressure induced vertigo was unexpected. This may be due to the low prevalence of patient-reported sound- and pressure-induced vertigo in our SCD population. We acknowledge that given the broad spectrum of symptom presentation, it may be difficult to capture symptomatology from chart review, which may be why the number of patient-reported sound- and pressure-induced vertigo is lower than that seen in the literature (12, 47). This highlights the need for a validated metric to measure symptom severity at presentation and post-intervention in order to more rigorously determine the association between physiological VEMP findings and symptomatology.

## Conclusion

Here we report the sensitivity and specificity of 4 kHz oVEMP amplitude in detecting clinically significant SCD_θ_. As previous studies have shown, we found that 4 kHz oVEMP n10 response alone performs better than 500 Hz oVEMP amplitude and cVEMP thresholds. We are able to further improve this detection by assessing 4 kHz oVEMP amplitude and proposing an optimal amplitude cut-off of 15 uV. Our results also suggest that 4 kHz oVEMP amplitudes align better with symptomatic SCD cases, compared to cases in which there is radiographic SCD but no characteristic symptoms. In situations in which there is radiographic evidence of dehiscence but the symptomatic presentation of patients is non-specific, a positive 4 kHz oVEMP can be useful in aiding clinicians in the diagnosis and management of SCD patients.

## Data Availability Statement

The raw data supporting the conclusions of this article will be made available by the authors, without undue reservation.

## Ethics Statement

The studies involving human participants were reviewed and approved by Stanford University Institutional Review Board (IRB-50573). Written informed consent for participation was not required for this study in accordance with the national legislation and the institutional requirements.

## Author Contributions

ET conducted data acquisition, analysis, and interpretation and made significant contributions to the design of the study, as well as the writing and editing of the manuscript. AS designed the audiometric database, conducted data acquisition, and was heavily involved in project design and manuscript editing. JS played a significant role in project design, data review and analysis, and manuscript editing and review. NB reviewed the data and was involved in the editing of the manuscript. MF contributed to the project design and manuscript review. KS led the conception of the study and contributed significantly to the data and manuscript review. All authors contributed to the article and approved the submitted version.

## Conflict of Interest

The authors declare that the research was conducted in the absence of any commercial or financial relationships that could be construed as a potential conflict of interest.
